# Implementation of Online Hospitals and Factors Influencing the Adoption of Mobile Medical Services in China: Cross-Sectional Survey Study

**DOI:** 10.2196/25960

**Published:** 2021-02-05

**Authors:** Huanlin Wang, LanYu Liang, ChunLin Du, YongKang Wu

**Affiliations:** 1 West China Hospital Sichuan University Sichuan China

**Keywords:** COVID-19, online hospital, mobile medical service, Unified Theory of Acceptance and Use of Technology, UTAUT

## Abstract

**Background:**

Online hospitals are part of an innovative model that allows China to explore telemedicine services based on national conditions with large populations, uneven distribution of medical resources, and lack of quality medical resources, especially among residents needing to be protected from COVID-19 infection.

**Objective:**

In this study, we built a hypothesis model based on the Unified Theory of Acceptance and Use of Technology (UTAUT) in order to analyze the factors that may influence patients’ willingness to use mobile medical services. This research was designed to assist in the development of mobile medical services. Residents who do not live in urban areas and cannot access medical assistance would greatly benefit from this research, as they could immediately go to the online hospital when needed.

**Methods:**

A cross-sectional study based at the West China Hospital, Sichuan University, was conducted in July 2020. A total of 407 respondents, 18 to 59 years old, in Western China were recruited by convenience sampling. We also conducted an empirical test for the hypothesis model and applied structural equation modeling to estimate the significance of path coefficients so that we could better understand the influencing factors.

**Results:**

Out of 407 respondents, 95 (23.3%) were aware of online hospitals, while 312 (76.7%) indicated that they have never heard of online hospitals before. Gender (*P*=.048) and education level (*P*=.04) affected people’s willingness to use online hospitals, and both of these factors promoted the use of online hospitals (odds ratio [OR] 2.844, 95% CI 1.010-8.003, and OR 2.187, 95% CI 1.031-4.636, respectively). According to structural equation modeling, the results of the path coefficient analysis indicated that performance expectancy, effort expectancy, and facilitating conditions have positive effects on patients’ willingness to use online hospitals.

**Conclusions:**

The goal of our research was to determine the factors that influence patients’ awareness and willingness to use online hospitals. Currently, the public’s awareness and usage of online hospitals is low. In fact, effort expectancy was the most important factor that influenced the use of online hospitals; being female and having a high education also played positive roles toward the use of mobile medical services.

## Introduction

### Background

With the advent of the *internet plus* era, information technology (IT) has brought great convenience to people's production and lives. At the same time, it has provided new ideas for medical service reform, and remote, mobile medical services have emerged [1]. As the largest developing country, China has a large population, an aging population, uneven distribution of medical resources, and lack of high-quality medical resources. Faced with increasing demand for medical and health services, around the year 2000, the Chinese government actively explored the application of internet IT to carry out innovative diagnoses and treatments based on the conditions seen nationwide. In this context, online hospitals have appeared, along with the emergence and spread of COVID-19, and were vigorously promoted to divert patients from hospitals and to reduce the risk of cross-infection in hospitals. The operation of online hospitals relies on offline physical medical institutions to provide patients with online follow-up services for common and chronic diseases through devices connected to the internet. Patients use mobile terminals to communicate with doctors online in the form of pictures, texts, voice messages, or videos [2]. Studies have shown that mobile medical services need to consider the public’s acceptance of these services; more attention should be paid to public awareness and experience during the adoption process in order to increase willingness to adopt services and increase utilization of services by users [3]. For one thing, online hospitals in China have just started to be implemented; as a result, some patients have little understanding of online hospitals. In addition, because of their traditional medical thinking, some patients have misunderstandings that lead them to believe that online hospitals are not reliable. Whether mobile medical services can be popularized and applied has become the focus of current research [4,5]. In order to explore patients’ willingness to use mobile medical services and their influencing factors, this study examines Chinese patients’ perspectives of online hospitals and builds a model of their willingness to use these services, based on the Unified Theory of Acceptance and Use of Technology. In this study, we discuss patients’ willingness to use mobile medical services of online hospitals and their influencing factors in order to provide strong evidence for the study of patients’ acceptance behavior of mobile medical services; in addition, using mobile medical methods to provide online diagnoses and treatment services for common and chronic diseases can be a reference point and offer suggestions for the global response to the COVID-19 outbreak.

### Literature Review and Hypotheses Development

In the field of IT, users’ technology adoption behavior has always attracted much attention. There are many models and theories that can be used to study user behavior, such as the technology acceptance model, the rational behavior theory, the planned behavior theory, and the UTAUT. The UTAUT is a self-rational action theory based on the technology acceptance model by Venkatesh and Davis and others [6,7]. This model is based on four structures: performance expectation, effort expectation, social influence, and convenience to explain the intention and behavior of individuals using technology [7]. Existing literature studies have shown that the UTAUT model provides better and more complete explanations about users’ technology adoption behavior than other technology acceptance models. This model not only promotes previous research results but it retains their simple structure. Therefore, it currently has the best predictive ability [8,9]. This model has been widely used in different fields of medical care. Research has confirmed that the UTAUT model is also acceptable in the field of health care mobile technology [10]. Phichitchaisopa and Naenna found that factors such as effort expectation, performance expectation, and facilitating conditions have a significant impact on the adoption of mobile health [11]. Cimperman et al used the UTAUT model to study mobile health factors in the elderly. They also found that performance expectation, effort expectation, social influence, technical anxiety, and other factors had a significant impact on users’ behavioral intentions [12]. Most of the relevant research in the field of mobile health, by both domestic and foreign scholars, focuses on product functions and use effects of mobile medical equipment and mobile health apps [13,14]; however, we have found no research on the willingness to use mobile online medical services. Moreover, mobile medical services of online hospitals are different from other mobile health care support applications. In their initial stage of promotion and operation, we should pay attention to the public’s expectations of, and willingness to use, these services [15,16]. Through the extension and application of the UTAUT model, we aim to determine the factors influencing willingness to use these services and the relationship between them; our research will fill this gap in the field of mobile medicine.

### Hypothetical Model Construction

Due to the special attributes of telemedicine, there will be some risk factors in the use process; for example, the mobile medical platform uploads patient information to the information system, patients and doctors need to communicate in a virtual environment, etc. The way these risks are perceived by the patient will affect their willingness to use the mobile medical platform [17]. Based on this and on the information system selected to adopt the UTAUT model, the *perceived risk* variable was added, which was used to analyze the patients’ perception of the risk of diagnosis and treatment via the internet. Finally, the hypothesis regarding patients’ willingness to accept medical treatment from online hospitals was formed. This study is based on the scales that have been researched and applied by scholars, both domestic and foreign, combined with the participation by online hospital patients. Through induction and summary, we have defined the model variables; the measurement items and model construction of each research variable in the model have also been proposed, as seen in Table 1 [18-27].

**Table 1:** Construct definitions and model assumptions.

Model variable	Definition	Model assumptions	References
Performance expectancy	Refers to the patients’ judgment of the benefit of mobile medical services	Performance expectancy has a positive impact on the patients’ willingness to use online hospitals	Venkatesh et al [18] Hoque and Sorwar [19] Hoque et al [20]
Effort expectancy	Refers to the patients’ judgment of the ease of acceptance and use of mobile medical services	Effort expectancy has a positive impact on the patients’ willingness to use online hospitals	Xue et al [21] Alalwan et al [22] Shareef et al [23]
Social influence	Refers to the external influence that patients perceive when using the online hospital app	Social influence has a positive impact on the patients’ willingness to use online hospitals	Nguyen et al [24] Hoque and Sorwar [19]
Perceived risk	Refers to the patients’ perception of possible adverse consequences through online hospital visits	Perceived risk has a negative impact on the patients’ willingness to use online hospitals	Kim and Park [25] Hsieh [26]
Facilitating conditions	Refers to whether the existing online hospital platform, technology, service-supporting measures, and personal equipment are sufficient to support mobile medical services	Facilitating conditions have a positive impact on the patients’ willingness to use online hospitals	Cilliers et al [27]

## Methods

### Participants

The average number of daily outpatients at the West China Hospital, Sichuan University, is about 16,000. Based on the fact that crowd gathering should be avoided during the COVID-19 pandemic, and considering the low awareness and utilization rates of online hospitals, the research team used convenience sampling to recruit survey subjects. Recruitment began on July 4, 2020, when questionnaires were distributed by specially trained investigators at the nurse stations of the West China Hospital, Sichuan University. The target group participants filled out the paper questionnaire on a voluntary basis or filled out the electronic questionnaire by scanning the code on their mobile phones. The questionnaires were answered anonymously and were collected on the spot. Inclusion criteria for participants included the following: (1) were between 18 and 59 years old; (2) were able to fill out the questionnaire independently, with clear consciousness and no obvious cognitive impairment; and (3) volunteered to participate in this study. Exclusion criteria included the following: (1) had a mental disorder and could not communicate normally and (2) refused to participate in this investigation.

### Design and Material

This is a cross-sectional study investigating patients’ willingness to use an online hospital platform. The research setting was a large general hospital in Western China. Internet diagnosis and treatment services involved in this study are closely related to internet use. A previous survey about online hospital participation by the elderly administered by our team found that most elderly people were not proficient in using mobile phone apps and had some difficulties operating mobile phones; the children of most of these participants helped them operate mobile phones and use the apps Therefore, this survey was mainly aimed at a sample of young and middle-aged participants.

### Procedure

Using the questionnaire survey method, the researchers designed their own questionnaires under the guidance of experts based on the UTAUT model, combined with the characteristics of online hospital operations. To ensure the validity of the questionnaire content, all measurement items were modified based on relevant domestic and foreign documents; see Multimedia Appendix 1 for details. The questionnaire used a 5-point Likert scale, ranging from 1 (completely disagree) to 5 (completely agree); the respondents chose the options that best suited their actual situations. Before the formal issuance of the questionnaire, the researchers conducted a presurvey; they selected 20 patients for the presurvey, modified the items in the questionnaire that were difficult to understand, and generated ambiguity. Finally, 31 of the most representative items of the questionnaire were selected. The data were collected from completed questionnaires and sorted using EpiData, version 3.1 (EpiData Association); the questionnaire data were statistically analyzed using SPSS Statistics for Macintosh, version 24.0 (IBM Corp), and SPSS Amos, version 24.0 (IBM Corp). The statistical methods were verified by descriptive analysis, reliability and validity tests, analysis of variance, and structural equation modeling, as needed.

## Results

### General Statistical Description

In this study, a total of 412 questionnaires were collected, of which 407 were valid, with an effective rate of 98.8%. The ratio of men to women in the survey was 1:1.19, including 319 young people out of 407 participants (78.4%) and 88 middle-aged people (21.6%). Among the survey respondents, 23.3% (95/407) knew about online hospitals, while 76.7% (312/407) said they did not know about, or had not heard of, online hospitals. A total of 95.3% (388/407) of the respondents expressed their support for, and had acceptable attitudes toward, the internet diagnosis and treatment model after being introduced to it by the researchers; see Table 2 for details.

**Table 2:** Participants characteristics.

Characteristic		Value (N=407), n (%)
**Gender**		
	Male	186 (45.7)
	Female	221 (54.3)
**Age (years)**		
	18-44	319 (78.4)
	45-59	88 (21.6)
**Education level**		
	Less than high school	16 (3.9)
	High school graduate	98 (24.1)
	Bachelor’s degree	193 (47.4)
	Master’s or doctoral degree	100 (24.6)
**Professional background**		
	Official	131 (32.2)
	Company employee	137 (33.7)
	Migrant worker	10 (2.5)
	Farmer	7 (1.7)
	Other	122 (30.0)
**Monthly income** **(RMB; US $1=RMB 6.4790)**		
	0-2000	40 (9.8)
	2001-5000	114 (28.0)
	5001-8000	127 (31.2)
	8001-10,000	68 (16.7)
	>10,000	58 (14.3)

### Reliability and Validity Test

This study used Cronbach α to measure the reliability of the questionnaire. The lowest value of Cronbach α for this questionnaire was .764 and the highest value was .900, indicating that the measurement scale had good reliability. In order to test the discriminant validity of variables in the model, we used SPSS Amos, version 24.0, to conduct confirmatory factor analysis on performance expectation, effort expectation, social impact, perceived risk, contributing factors, and willingness to use the platform. The indicators were as follows: adjusted goodness-of-fit index (AGFI)=0.858, goodness-of-fit index (GFI)=0.893, comparative fit index (CFI)=0.935, root mean square error of approximation (RMSEA)=0.063, composite reliability (CR)>0.7, and average variance extracted (AVE)>0.5. It can be seen that the goodness of fit of the model was good; that is, the variables had good discriminant validity (see Tables 3 and 4).

**Table 3:** Results of the questionnaire reliability analysis.

Dimension and item number		Correlation matrix between items				Corrected item’s total correlation	Cronbach α if item deleted	Cronbach α of the dimension
**Performance expectancy (PE)**								.784
	PE.1	1				0.538	.895	N/A^a^
	PE.2	0.715	1			0.647	.893	N/A
	PE.3	0.413	0.448	1		0.452	.898	N/A
	PE.4	0.403	0.489	0.614	1	0.598	.894	N/A
**Effort expectancy (EE)**								.764
	EE.1	1				0.690	.891	N/A
	EE.2	0.576	1			0.673	.892	N/A
**Social influence (SI)**								.802
	SI.1	1				0.550	.895	N/A
	SI.2	0.629	1			0.572	.894	N/A
	SI.3	0.566	0.660	1		0.502	.896	N/A
	SI.4	0.415	0.429	0.382	1	0.699	.891	N/A
**Facilitating conditions (FC)**								.875
	FC.1	1				0.701	.891	N/A
	FC.2	0.638	1			0.663	.892	N/A
	FC.3	0.581	0.659	1		0.651	.893	N/A
	FC.4	0.630	0.586	0.727	1	0.675	.892	N/A
**Perceived risk (PR)**								.841
	PR.1	1				0.234	.903	N/A
	PR.2	0.600	1			0.225	.904	N/A
	PR.3	0.525	0.633	1		0.227	.904	N/A
	PR.4	0.473	0.558	0.646	1	0.140	.909	N/A
**Behavioral intention (BI)**								.879
	BI.1	1				0.666	.892	N/A
	BI.2	0.662	1			0.631	.893	N/A
	BI.3	0.629	0.734	1		0.625	.893	N/A
Total scale								.900

^a^N/A: not applicable; Cronbach α in this column was calculated for each dimension, not for each item.

**Table 4:** Results of the questionnaire validity analysis.

Dimension and item number		Standardized factor load	Composite reliability	Average variance extracted^a^
**Performance expectancy (PE)**				0.693
	PE.1	0.684	0.899	N/A^b^
	PE.2	0.832		N/A
	PE.3	0.933		N/A
	PE.4	0.862		N/A
**Effort expectancy (EE)**				0.522
	EE.1	0.625	0.710	N/A
	EE.2	0.695		N/A
**Social influence (SI)**				0.681
	SI.1	0.868	0.864	N/A
	SI.2	0.845		N/A
	SI.3	0.758		N/A
**Perceived risk (PR)**				0.550
	PR.1	0.867	0.826	N/A
	PR.2	0.804		N/A
	PR.3	0.541		N/A
	PR.4	0.714		N/A
**Facilitating conditions (FC)**				0.617
	FC.1	0.780	0.828	N/A
	FC.2	0.695		N/A
	FC.3	0.872		N/A
**Behavioral intention (BI)**				0.567
	BI.1	0.568	0.793	N/A
	BI.2	0.829		N/A
	BI.3	0.831		N/A

^a^The fitting indices were as follows: χ^2^/df=2.1, adjusted goodness-of-fit index (AGFI)=0.858, goodness-of-fit index (GFI)=0.893, comparative fit index (CFI)=0.935, and root mean square error of approximation (RMSEA)=0.063.

^b^N/A: not applicable; average variance extracted was calculated for each dimension, not for each item.

### Structural Equation Model Verification

**Figure 1:**
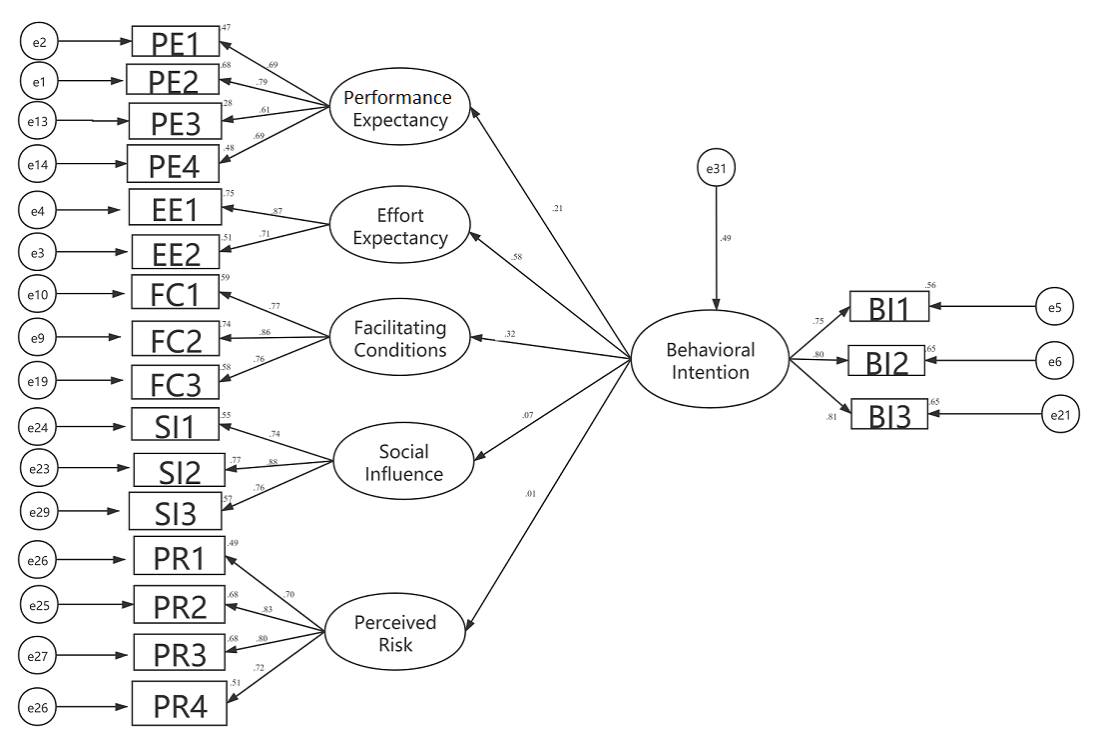
Results of the structural equation model analysis. Note, e1 to e31 are residuals, which are not explained in the hypothesis equation. The number on each arrow indicates the factor load of the latent variable on the observed variable: the larger the factor load, the better. If the value is greater than .40 and less than 1, the contribution is qualified.

Structural equation modeling is a statistical method used to analyze the relationship between variables. According to the degree of consistency between the theoretical model and the actual data, the theoretical model is evaluated to achieve the goals of quantitative research on actual problems. This method overcomes the shortcomings of SPSS software’s widely used multiple regression analysis method. It not only explains the relationship between variables but also allows the existence of measurement error of the variables. It can realize the estimation of factor structure and relationship as well as the simultaneous estimation of the degree of model fitting. In this study, the structural equation model analysis software SPSS Amos, version 24.0, was used to draw the structural equation model analysis diagram (see Figure 1); the model verification of the parameters of the initial hypothetical model was carried out to analyze the relationship between the variables and their influence mechanisms (see Table 5). From the significance of the model path coefficient, it can be seen that Hypothesis 1, Hypothesis 2, and Hypothesis 5 are valid, while the assumptions of Hypothesis 3 and Hypothesis 4 are not valid. That is to say, performance expectation, effort expectation, and contributing factors have significant effects on patients’ willingness to use mobile medical services. However, social influence and perceived risk do not have significant effects on patients’ willingness to use mobile medical services. In order to ensure the rigor of the model’s logic, the two insignificant paths mentioned above were deleted, the revised model was obtained, and the SPSS Amos, version 24.0, program was run again to verify each parameter. The corrected significance level of each path coefficient was less than 0.001, and the model-fitting indexes were as follows: χ^2^/df=3.5, CFI=0.899, AGFI=0.900, and RMSEA=0.092. These values indicate that the revised model fit was acceptable.

According to the estimation result of the model path coefficient and the analysis result of the adjustment effect, we finally determined the following: performance expectation, effort expectation, and contributing factors are the three main factors influencing patients’ use of online hospitals and they play a positive role.

**Table 5:** Verification of the path coefficients of the initial hypothesis model.

Hypothesis (H)	Path	Estimate	SE	Composite reliability	*P* value	Results
H1	PE^a^→BI^b^	0.21	0.063	2.587	.01	Established
H2	EE^c^→BI	0.58	0.076	6.290	<.001	Established
H3	SI^d^→BI	0.07	0.045	1.217	.22	Not established
H4	PR^e^→BI	0.01	0.031	0.286	.78	Not established
H5	FC^f^→BI	0.32	0.055	4.414	<.001	Established

^a^PE: performance expectancy.

^b^BI: behavioral intention.

^c^EE: effort expectancy.

^d^SI: social influence.

^e^PR: perceived risk.

^f^FC: facilitating conditions.

### The Relationship Between Different Demographic Characteristics and Willingness to Use Online Hospitals

Structural equation modeling and hypothesis tests were applied in this research. A *P* value less than .05 is considered statistically significant. To measure the relationship between gender, age, education level, income, and willingness to use online hospitals, we conducted a separate test by establishing a logistic regression model, and the result is summarized in Table 6. Here, we took *male* gender and *less than high school* education level as the reference group and transformed behavioral intention into a binary variable (0: reject; 1: accept). We found that gender (*P*=.048) and education level (*P*=.04) affected the willingness to use online hospitals, and both of them promoted the use of online hospitals (odds ratio [OR] 2.844, 95% CI 1.010-8.003, and OR 2.187, 95% CI 1.031-4.636, respectively) as shown in Table 6. In other words, female participants’ willingness to use online hospitals was 2.844 times that of male participants, while willingness to use online hospitals increased 2.187 times (ie, OR 2.187, 95% CI 1.031-4.636) with each level of education.

**Table 6:** Variables in the equation.

Variable	B	SE	Wald value	*P* value	Odds ratio (95% CI)
Gender	1.045	0.528	3.918	.048	2.844 (1.010-8.003)
Age	–0.262	0.444	0.349	.56	0.769 (0.323-1.836)
Education	0.782	0.383	4.162	.04	2.187 (1.031-4.636)
Income	–0.128	0.219	0.343	.56	0.880 (0.573-1.351)

## Discussion

### Principal Findings

A total of 407 young and middle-aged patients at a hospital in Western China were surveyed regarding their willingness to use an online hospital. This study analyzed the factors, and their relationships, that influenced patients’ use of online hospitals and provided some prior information and a theoretical basis upon which other researchers can carry out similar research. The results show that the public’s awareness and usage of online hospitals was low. According to the estimation results of the model path coefficient and the analysis of the adjustment effect, we determined the following: performance expectancy, effort expectancy, and facilitating conditions were the three main factors that affected patients’ willingness to use an online hospital. They all had a positive impact on behavioral intention. It can also be seen from the path coefficient results in Table 5 that effort expectancy is the most important factor. This may indicate that the user’s effort expectancy shows the user’s ability to accept new things to a certain extent. When users encounter unfamiliar content when operating within the online hospital platform, their efforts will inspire them to try and learn new things, so as to make better use of online hospitals. From Table 5, we can see that performance expectancy had a positive impact on behavioral intention, which shows that perceived ease of use of mobile medical platforms and the usefulness of mobile medical services may have a positive effect on patients’ willingness to use online hospitals [28]. Furthermore, facilitating conditions also had a positive impact on behavioral intention, which shows that some external promotion effects, such as medical staff recommendations and guidance, government policy support, and policies that benefit people, will probably increase the willingness of patients to use online hospital mobile medical services. In addition, gender and education level were personal factors that influenced patients’ willingness to use mobile medical services. It can be seen from the OR value in Table 6 that women were more likely to accept mobile medical services of online hospitals than were men; the acceptability by women was 2.844 times that of men. Meanwhile, the higher the education level that a patient had, the more adventurous the patient became. The acceptance increased 2.187 times with each increase in education level. People with higher education levels may be more willing to try this new type of technology platform. Throughout the whole process, it was indicated that certain patient attributes may influence people’s decisions. Further research is needed to explore these relationships more deeply.

### Research Impact

The current outbreak of COVID-19 is still spreading all over the world, which poses great threats to the safety and health of people around the world. Medical systems in all countries are facing great amounts of pressure and challenges. This study has several implications for both researchers and practitioners of mobile medicine and provides a reference for the response to the COVID-19 pandemic. Online hospitals constitute a new mode of telemedicine. Members of the public are limited by traditional modes of medical thinking and their awareness is relatively low. Most of them hold a conservative wait-and-see attitude. There will be a certain resistance to this in the early stages. Medical habits and thinking styles require a transformation process [29]. To change the public’s resistance to change, the government and medical institutions need to continue to increase the promotion of mobile medical services. It will be important to pay attention to improving public eHealth literacy [30], especially among middle-aged and elderly groups who know little about new technologies and have relatively low levels of education. Differentiated promotion in different age groups will be important, focusing on the interaction between patient groups. At the same time, it is necessary to fully standardize the management of mobile medical practitioners; strictly control the quality of mobile medical services; conduct daily monitoring and scoring of doctors’ qualification reviews, prejob training, medical quality, service attitudes, and patient satisfaction; and establish multidimensional online assessment standards and scientific management of every aspect of mobile medical service. In this regard, government departments should play a leading role. We can learn from the experience of developed countries with regard to the supervision of mobile medical services [31,32]. It will be important to accelerate the improvement of relevant legislation and regulatory policies and to formulate mobile medical service quality standards, industry regulations, and information security standards in order to promote the long-term development of online hospitals.

### Research Limitations

Since this study recruited a cross-sectional sample of young and middle-aged participants in Western China, it may not truly reflect the willingness of the entire Chinese population or groups in other regions to adopt mobile medical services. In addition, this study found that social influence and perceived risk have no significant impact on patients’ willingness to use online hospitals. This is inconsistent with the results of other studies on the willingness to adopt other mobile health technologies. Further research is needed to re-examine social influence and perceived risk. At the same time, mobile medical services are under continuous development in China, and the relevant research has not yet formed a relatively complete research framework.

## Appendix

Multimedia Appendix 1 Measurement items of research variables.

## Abbreviations

AGFI: adjusted goodness-of-fit index
AVE: average variance extracted
CFI: comparative fit index
CR: composite reliability
GFI: goodness-of-fit index
IT: information technology
OR: odds ratio
RMSEA: root mean square error of approximation
UTAUT: Unified Theory of Acceptance and Use of Technology
