# Supplementary material for: Implementation of Online Hospitals and Factors Influencing the Adoption of Mobile Medical Services in China: Cross-Sectional Survey Study
Source: JMIR Mhealth Uhealth. 2021 Feb 5;9(2):e25960. doi: 10.2196/25960 (PMC7869921; doi:10.2196/25960)
Supplement: Multimedia Appendix 1 [file mhealth_v9i2e25960_app1.docx]

\

Measurement items of research variables

| variable | Serial number | Measurement items |
| --- | --- | --- |
| Performance Expectancy | PE1 | The use of Online hospital mobile medical platform can reduce queue registration and round trip time, and improve the efficiency of medical treatment. |
|  | PE2 | Using the Online hospital mobile medical platform, I can interact with doctors more conveniently without the limitation of time and region. |
|  | PE3 | Compared with the hospital face-to-face treatment, I think Online doctors can provide the same quality of diagnosis and treatment services |
|  | PE4 | Through the Online hospital mobile medical platform, I can obtain personalized health management (such as family doctor signing) |
| Effort  Expectancy | EE1 | I think it's easy for me to accept Online diagnosis and treatment |
|  | EE2 | It is easy for me to learn and use mobile medical app platform (such as Online hospital) |
| Social  Influence | SI1 | The recommendation of medical professionals will affect my use of Online hospital mobile medical platform |
|  | SI2 | The government’s policy guidance and incentive measures (such as medical insurance reimbursement, etc.) will affect my use of the Online hospital platform |
|  | SI3 | The experience of people around me using the Online hospital will affect my use of the Online hospital platform |
|  | SI4 | Online diagnosis and treatment is the trend of the times. I will keep pace with the times and use the Online hospital platform |
| Facilitating  Conditions | FC1 | I have sufficient conditions / equipment (smart phone and other terminal equipment, stable network) to use Online hospital platform |
|  | FC2 | Compared with the traditional face-to-face treatment, the cost is economical and reasonable, which will promote me to use the Online hospital platform |
|  | FC3 | The operation mode of Online hospital platform is clear and easy to understand, which helps me to use it better |
|  | FC4 | Provide detailed operation instructions and manual consultation, so that I can better use the Online hospital platform |
| Perceived  Risk | PR1 | I'm worried about the qualification of online doctors and have scruples about the accuracy of diagnosis and treatment results |
|  | PR2 | I'm worried about the risk of personal privacy leaking online |
|  | PR3 | If there is a medical dispute, I'm afraid I can't protect my personal rights and interests |
|  | PR4 | I'm worried that the quality and distribution of drugs can't be guaranteed |
| Behavioral  Intention | BI1 | I am willing to learn how to use the mobile Online hospital platform |
|  | BI2 | I am willing to use the mobile Online hospital platform for online consultation of common diseases and chronic diseases |
|  | BI3 | I would like to recommend the Online hospital diagnosis and treatment service to my family and friends |
